# Seroepidemiological analysis of toxoplasmosis in college students

**DOI:** 10.1186/1678-9199-21-1

**Published:** 2015-01-05

**Authors:** Jaqueline Polizeli Rodrigues, Fernando Frei, Italmar Teodorico Navarro, Luciana Pereira Silva, Monica Yonashiro Marcelino, Heitor Franco de Andrade-Junior, Carolina Arruda de Faria, Marislene Santos, João Tadeu Ribeiro-Paes

**Affiliations:** Department of Biological Sciences, (UNESP), Assis, São Paulo State Brazil; Department of Veterinary Medicine, Londrina State University, Londrina, Paraná State Brazil; Graduate Program in Biotechnology, University of São Paulo (USP)/Butantan Institute/Institute for Technological Research (IPT), São Paulo, São Paulo State Brazil; Institute of Tropical Medicine of São Paulo, University of São Paulo (USP), São Paulo, São Paulo State Brazil; Department of Genetics, Ribeirão Preto Medical School, University of São Paulo (USP), Ribeirão Preto, São Paulo State Brazil; Laboratório de Genética e Terapia Celular (GenTe CeL), Departamento de Ciências Biológicas, Universidade Estadual Paulista (UNESP), Campus de Assis, Av. Dom Antônio, 2100, Assis, SP CEP 19.806-900 Brazil

**Keywords:** *Toxoplasma gondii*, Toxoplasmosis, Enzyme-linked immunosorbent assay, Indirect fluorescent antibody test, Seroprevalence, Epidemiology

## Abstract

**Background:**

Toxoplasmosis is a zoonosis caused by an obligate intracellular parasite, *Toxoplasma gondii*, which affects warm-blooded animals including humans. Its prevalence rates usually vary in different regions of the planet.

**Methods:**

In this study, an analysis of the seroprevalence of toxoplasmosis among Brazilian students was proposed by means of IgG specific antibodies detection. The presence of anti-*Toxoplasma gondii* antibodies by indirect fluorescent antibody test (IFAT) was also evaluated in order to compare it with enzyme-linked immunosorbent assay (ELISA) and to assess the use of 2,2′-azinobis(3-ethylbenzothiazoline-6-sulfonic acid) and o-phenylenediamine dihydrochloride chromogens.

**Results:**

The IFAT method showed a seroprevalence of 22.3%. These results were similar to those obtained by ELISA (24.1%). The seroprevalence was directly estimated from the IgG avidity, which showed that in a sample of 112 students, three of them had acute infection, an incidence of 1.6% in the studied population.

**Conclusion:**

In this study, the use of different chromogenic substrates in immunoenzymatic ELISA assays did not display different sensitivity in the detection of *T. gondii*-reagent serum. The extrapolation of results to this population must be carefully considered, since the investigation was conducted on a reduced sample. However, it allows us to emphasize the importance of careful and well prepared studies to identify risk factors for toxoplasmosis, to adopt preventive measures and to offer guidance to at-risk populations about the disease.

## Background

*Toxoplasma gondii* is a protozoan parasite that is responsible for toxoplasmosis in warm-blooded animals [1–7]. The infection is spread mainly through contact with oocysts eliminated in feces of infected cats, ingestion of contaminated raw or undercooked meat and congenital infection [1, 8–14]. The environmental contamination by this protozoa demands attention since it may trigger other routs of transmission and can lead to outbreaks, such as the waterborne epidemic in Santa Isabel do Ivaí, Paraná state, Brazil (23° 0′ 19″ S, 53° 11′ 16″ W) [15].

Human infection is usually asymptomatic. The main clinical signs after the onset of the disease include lymphadenitis, fever, asteny, and myalgia. Encephalitis, meningoencephalitis, ocular lesions, septic syndrome, myocarditis or hepatitis may be found occasionally [1, 3, 16, 17]. Symptoms are manifested mainly in immunocompromised people and newborns [18–21].

Recently the diagnosis of toxoplasmosis has been drawing close attention. Several serological techniques have been applied and have shown good sensitivity, specificity, and are quickly carried out. Among different diagnostic techniques, the following are noteworthy: Sabin and Feldman technique, indirect fluorescent antibody test (IFAT), complement fixation (CF), hemagglutination (HA), enzyme-linked immunosorbent assay (ELISA) and immunosorbent agglutination assay (ISAGA) [22–27].

Seroprevalence indexes have varied in different countries and regions of the planet [28]. Serological research showed rates of 6.7% in Korea, 12.3% in China, 22.5%, in the US adult population, 23.9% in Nigeria, 38.8% in Spain, 46% in Tanzania and 47% in rural zones of the France [29–35]. A study with anti-*T. gondii* antibodies carried out in the Republic of Benin revealed a seroprevalence of 87.7% [36]. In different regions in Brazil, the seropositive rate varied between 37% and 91% [37, 4].

Three suspicious cases of toxoplasmosis were reported in São Paulo State University (UNESP), in Assis, São Paulo state, Brazil (22° 39′ 42″ S, 50° 24′ 44″ W) stirring up a series of controversies and discussions. The first one refers to clinical likelihood of the disease in college students. However, no serological test was conducted to uphold the diagnosis. The second one is the relation between these cases was the increased cat population. And, finally, a student who had an eye affliction and tested positive for *T. gondii*.

Since these reports and suppositions were based on inconsistent diagnostic assertions and subjective causal links, without the due clinical evaluation and laboratory diagnostic evidence, a project was devised to find out the seroprevalence and incidence of toxoplasmosis in a sample of UNESP students, by means of serological tests for IgG specific antibodies detection. In addition, the study also verified the presence of anti-*T. gondii* antibodies in students by IFAT, in order to compare IFAT and ELISA techniques for the toxoplasmosis diagnosis, and to evaluate the use of 2,2′-azinobis(3-ethylbenzothiazoline-6-sulfonate) (ABTS) and o-phenylenediamine dihydrochloride (OPD) chromogens in the ELISA test for the toxoplasmosis diagnosis.

## Methods

The project was carried out at the Laboratory of Human Parasitology and Immunology of São Paulo State University (UNESP), in Assis, with the cooperation of the Laboratory of Zoonoses and Public Health of the Department of Preventive Veterinary Medicine, State University of Londrina (UEL), in Londrina, Paraná state, Brazil (23° 19′ 39.5″ S, 51° 11′ 56.5″ W) and of the Laboratory of Protozoonoses of the Institute of Tropical Medicine of São Paulo, University of São Paulo (USP), São Paulo, São Paulo state, Brazil (23° 33′ 18.5″ S, 46° 40′ 18.2″ W).

### Characteristics of the area and population under study

**Figure 1:**
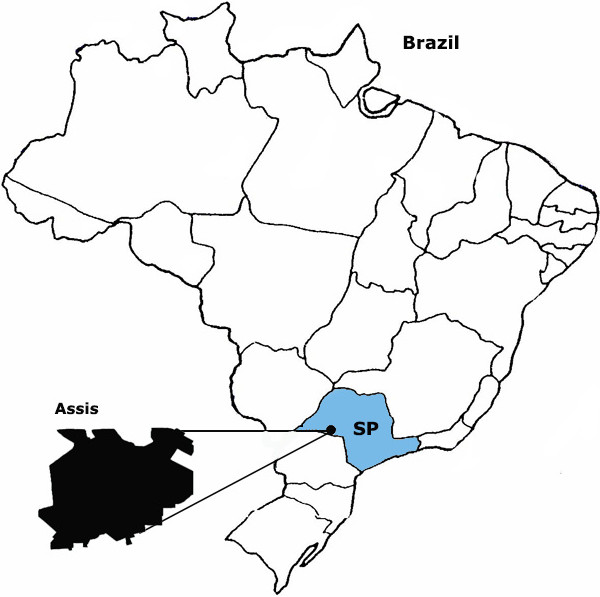
**Assis is located in the west region of São Paulo state, Brazil, 434 km away from the state capital and comprises a population of 100.911 inhabitants** [38]**.** The annual average temperature is 21.37°C.

Assis is located in western São Paulo state, Brazil, 434 km away from the state capital and comprises a population of 100.911 inhabitants. The annual average temperature is 21.37°C (Figure 1) [38].

Serological tests were carried out in 112 UNESP freshmen of Assis campus, within an age range from 17 to 25 years old. The selection of students was random (by a raffle) and the sample size was proportional to the number of students in each course (Table 1). Freshman students answered a questionnaire, in order to establish the likelihood of epidemiological correlations, risk factors and contact with cats before being accepted into the university.

**Table 1:** **Distribution of new students per course in 2009 in UNESP campus of Assis, and sample size of subjects who provided biological material (blood) for serological analysis**

Course	Freshman	Blood samples
Biology	40	20
Biotechnological engineering	40	17
History	40	19
Psychology	90	29
Languages	80	27
Total	290	112

### Collection of blood and serum samples

The serological information of the students was recorded in individual files and kept confidential. Two blood samples were collected from each voluntary subject of the research. The first one was immediately after the students arrived for the beginning of the 2009 school year and the second one, six months after the first sample had been collected. A 5-mL blood sample was collected from each student, by brachial venous puncture. Meticulous aseptic cautions were taken for blood collection and for the protection of those in charge of it. The samples were sent to the Laboratory of Human Parasitology and Immunology for blood processing, plasma separation and storage for later serological analysis methods. Each serum sample was obtained from the whole blood, after centrifugation at 350 × *g*, for 15 minutes. The resulting supernatant was stored at −20°C.

### Indirect Fluorescent Antibody Test (IFAT)

The indirect fluorescent antibody test (IFAT) technique for the screening of anti-*T. gondii* antibodies, of the IgG class, was carried out according to the methodology described by Camargo [23]. Human anti-IgG serum produced from rabbit inoculations was employed conjugated with fluorescein isothiocyanate (Sigma Chemical Corp., USA). For each serum sample, dilutions at 1:16, 1:64, 1:256, 1:1024 and 1:14096 were prepared. All reactions had a previously known positive and negative control serum. The reading of the plates was made in a fluorescence microscope (Nikon™, Japan). Dilutions greater than or equal to 1:16A were considered positive results.

### ELISA immunoenzymatic test

Anti-*T. gondii* IgG antibodies were determined by the methodology of Camargo [24] and Uchôa *et al*. [39]. Plates with flat-bottom wells (Corning®, USA) sensitized with 1 μg/mL of *T. gondii* soluble antigen extract were used for OPD ELISA; and those sensitized with 10 μg/mL of *T. gondii* soluble antigen extract were used for ABTS ELISA. Plates were sensitized with antigen by incubation at 4°C for 18 hours, with 100 μL of antigen solution per well. After washing, plates were blocked by incubation for one hour at 37°C with 250 μL of PBS-T per cavity (0.05% of Tween 20 in phosphate-buffered saline solution – PBS) containing 3 g of skimmed milk powder (Molico®, Nestlé, Brazil). Afterward, plates were washed five times in PBS-T for five-minute periods. Plates containing serum with human-peroxydase-conjugated anti-IgG dilutions (Sigma®, USA) were incubated for the test in stove for one hour. Following both incubations, plates were washed three times in PBS-T for five minutes. The color reaction was carried out subsequently in the dark for 15 minutes at room temperature with 100 μL of the OPD chromogenic solution (o-phenylenediamine 0.05%, Sigma® + citric acid 1% + Na_2_HPO_4_ 1.45% in H_2_O, addition of 10 μL de H_2_O_2_ 30% for each 20 mL of the solution) and was quenched by the addition of 25 μL of 2 M sulfuric acid solution. For the OPD chromogenic solution, the optical density (OD) was determined by the reading at 492 nm, and for the ABTS chromogenic solution, by the reading at 414 nm and 612 nm, in a plate spectrophotometer (700 Plus Spectrophotometer, Fendo, Brazil). For OPD ELISA and ABTS ELISA, in both the first analysis and the one six months after, the cutoff point was obtained from the mean of results from negative control sera evaluated by IFAT plus two standard deviations.

### IgG avidity

The same procedure was used for IgG analysis through in-house ELISA, according to the methodology described by Hedman *et al.* [40]. The plate was coated by the addition, for 24 hours at 4°C, of 100 μL of *T. gondii* protein antigen to the wells at 1 μg/mL concentration, in an 8 M urea chaotropic (Sigma®) solution in PBS. After blocking possible free sites, the wells received 100 μL of PBS-T diluted serum samples (1/100, 1/200, 1/400, 1/800). Plates were incubated for one hour at 37°C and washed five times in PBS-T. For each dilution, two wells received additional ten-minute incubation at 37°C with 6 M urea neutral chaotropic solution (Sigma®) in PBS-T. Wells containing the control solutions were kept in PBS-T. Afterwards, the antibody development was carried out with the use of human anti-IgG peroxydase conjugate (Sigma®).

The estimation of effective titer of each serum was carried out by means of the isolated dilutions of each sample in a log-log linear regression model. The titers of total or high avidity chaotropic-resistant antibodies were achieved by means of log-log regression, with the use of the values able to generate 1.0 absorbance in ELISA (Log = 0). Avidity (AVT) was determined by the percentage of chaotropic resistant titers. Samples with AVT values higher than 30% were considered as high avidity. The optical density (OD) was determined by the reading at 492 nm in a plate spectrophotometer.

### Statistical analysis

The correlation rate was used for the analysis among the interspecific titers and the analysis of association among the positive results and variables related to risk factors (gender, direct contact with soil, contact with cats, consumption of raw or undercooked meat and raw vegetables). Values were submitted to the chi-square analysis, with the help of the Epi Info version 6.04 statistics software [41]. A reliable 95% range was considered and p-value < 0.05 was considered statistically significant. In order to check the agreement among ELISA and OPD or ABTS chromogen and IFAT diagnosis tests, the results were reviewed by the kappa test (K), and by sensitivity, specificity, positive predictive value and negative predictive value. Statistical comparisons were made using the GraphPad Prism version 5.00 software for Windows (Graphpad Software, USA).

### Ethics committee approval

The present study was approved by the Research Ethics Committee of Marília Medical School (FAMEMA – Marília, SP, Brazil) under protocol n. 449/08. It included only patients who authorized the collection of blood samples through free informed consent according to the Brazilian National Committee of Ethics in Research (CONEP), resolution 236/96, National Health Council, Brazilian Ministry of Health.

## Results

Serum samples from 112 students were submitted to anti-*T. gondii* IgG antibody screening by means of IFAT and ELISA with OPD or ABTS chromogen substrate. The correlation between the results obtained in the immunoenzymatic and immunofluorescence assays may be seen in Table 2, in which two discordant results can be noted, two samples that tested positive by OPD ELISA were negative by IFAT whereas two other samples that were positive by ABTS ELISA tested negative by IFAT.

**Figure 2:**
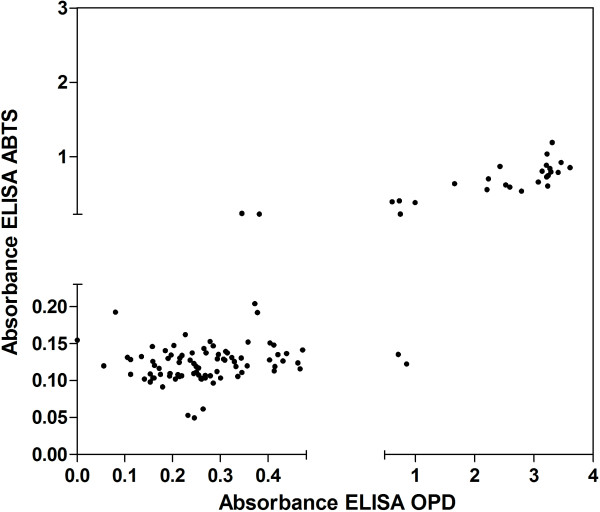
**Quantitative reactivity and linear regression analysis of IgG anti-** ***T. gondii*** **in 112 serum samples from university students using ELISA with chromogen substrate OPD and ABTS.** Interruptions in the axes show the thresholds of positivity of the assays (ELISA OPD cutoff = 0.486 and ELISA ABTS cutoff = 0.239).

The comparison between OPD and ABTS ELISA enzymatic assays showed high agreement (kappa = 0.9024), 92.5% sensitivity (95% confidence interval – CI: 90.5-96.3) and 97.6% specificity (95% CI: 93.2-97.8) and the positive predictive values were 92.5% (95% CI: 90.6-96.3) and negative predictive values 97.6% (95% CI: 93.2-97.8) (Figure 2).

**Table 2:** **Results of anti-** ***T. gondii*** **in serum samples of 112 UNESP students by means of IFAT (reference test), ELISA with OPD or ABTS chromogen substrate**

Assay	Result	IFAT	
		IgG	
		Positive	Negative
ELISA IgG	Positive	25	2
OPD	Negative	0	85
K = 0.949			
ELISA IgG	Positive	25	2
ABTS	Negative	0	85
K = 0.949			

ABTS: 2.2′-azinobis(3-ethylbenzothiazoline-6-sulfonate), CI: confidence interval, ELISA: enzyme-linked immunosorbent assay, IFAT: indirect fluorescent antibody assay, IgG: immunoglobulin G, K: kappa index, OPD: O-phenylenediamine dihydrochloride.

According to IFAT, the usual standard, the seroprevalence was 22.3% (95% CI: 14.5-28.7), similar to the one found in two imunoenzymatic tests. The OPD ELISA test showed a seroprevalence of 24.1% (95% CI: 16.2-32.0), the same found in the ABTS ELISA.

The confirmation and comparison of IgG serological test results by IFAT and ELISA with OPD and ABTS chromogen substrate methods are shown in Table 2. There is a high agreement among the three methods. The comparison of IFAT with OPD and ABTS ELISA test showed a higher agreement than the one found among the immunoenzymatic tests alone (kappa = 0.9499) with 100% of sensitivity (95% CI: 97.6%-100), specificity of 97.7% (95% CI: 94.2-99.1), positive predictive value of 92.5% (95% CI: 88.6-93.8), and negative predictive value of 100% (95% CI: 96.7-100).

The use of different chromogenic substrates in the immunoenzymatic ELISA assays did not show different sensitivity for detecting *T. gondii*-reagent serum. The ELISA test with OPD chromogenic substrate with plates sensitized with 1 μg of antigen revealed 27 positive sera, the same result observed in the ELISA IgG test with ABTS chromogenic substrate and plates sensitized with 10 μg of antigen.

After six months, new blood samples were examined, by means of IFAT and ELISA with OPD and ABTS chromogen substrates, in order to check the presence of IgG anti-*T. gondii* antibodies. The analyses of 112 students showed that three who tested negative in the first time, six months after were seropositive by IFAT, OPD ELISA and ABTS ELISA. The other serum samples showed the same results of first analysis, with exception of four samples: two were negative by IFAT and OPD ELISA and positive by ABTS ELISA whereas the other two were negative by IFAT and ABTS ELISA and positive by OPD ELISA. The three seroconverted samples were submitted to IgG avidity test. Students showed low IgG avidity, indicating recently acquired infection (Table 3).

**Table 3:** **Serological profile of three UNESP students in Assis six months after the first analysis**

Age (years)	IgG ^a^ ELISA OPD (OD)	IgG ^b^ ELISA ABTS (OD)	IFAT IgG	AVT (%)	Interpretation ^c^	Serological profile
23	0.491	0.069	Positive	28.90%	Low avidity	Acute phase
20	2.440	0.646	Positive	27.41%	Low avidity	Acute phase
21	3.372	0.851	Positive	29.65%	Low avidity	Acute phase

^a^cutoff: 0.427; ^b^cutoff: 0.054; ^c^IgG low avidity < 30%.

AVT: avidity of titer, ELISA: enzyme-linked immunosorbent assay, IgG: immunoglobulin G, OD: optical density.

In the present study, the seroprevalence of toxoplasmosis was 22.3% and 25% in the first and second evaluation, respectively. The incidence was directly estimated from the IgG avidity, which showed that, from the 112 students, three had results suggesting acute infection with an incidence of 1.6% (95% CI: 0.6-4.7%), which allows projecting an annual incidence of 3.2% in the studied population.

Table 4 shows the distributions of the variables according to *T. gondii* infection frequency. No differences were observed when variables taken into account were: gender (χ2 = 0.23, p > 0.05), contact with the cats of the university (χ2 = 1.4; p > 0.05), consumption of raw vegetables (χ2 = 0.11; p > 0.05), and the direct contact with soil (χ2 = 0.58, p > 0.05). The variables contact with cats outside the university (χ2 = 4.2; p < 0.05) and ingestion of raw or undercooked meat (χ2 = 5.0, p < 0.05) were the statistically significant.

**Table 4:** **Anti-** ***T. gondii*** **IgG antibody levels in students by means of indirect fluorescent antibody test (IFAT), and risk factors for toxoplasmosis**

Risk factors	Total	Anti-***T. gondii***IgG positive	Anti-***T. gondii***IgG negative	χ2 (p values)
Gender % of males (n/total)	40.1% (45/112)	36.0% (9/25)	41.3% (36/87)	0.23 (NS)
Direct contact with soil (n/total)	1.8% (2/112)	0% (0/25)	2.2% (2/87)	0.58 (NS)
Contact with the cats of the university (n/total)	4.4% (5/112)	0% (0/25)	5.7% (5/87)	1.4 (NS)
Contact with cats outside the university (n/total)	26.9% (29/112)	76.0% (23/25)	11.4% (10/87)	4.2 (p < 0.05)
Eating raw or undercooked meat (n/total)	33.0% (37/112)	92.0% (23/25)	16.0% (14/87)	5.0 (p < 0.05)
Raw vegetables consumption (n/total)	94.6% (106/112)	96.0% (24/25)	94.2% (82/87)	0.11 (NS)

χ2: chi square test; NS: not significant (p > 0.05).

## Discussion

The screening of IgG anti-*T. gondii* antibodies by IFAT showed a 22.3% prevalence in students. The seroprevalence data achieved by IFAT serological method were confirmed by the immunoenzymatic ELISA assay with OPD and ABTS chromogenic substrate (24.1%). The tests had highly coherent results, an unusual fact for assays with different antigens such as IFAT and hemagglutination [24].

Therefore, as proposed by Tanyuksel *et al.* [42] and Reis *et al.* [43], the avidity test was employed to identify cases suggesting recent infection. The results enabled the projection of an annual seroconversion of 3.2%. Although the avidity test may help to explain the *Toxoplasma gondii* acute infection diagnosis, some patients have shown persistent low avidity antibodies against the lysate of *T. gondii* antigenic cells for several months [44, 45]. On the other hand, IgG antibody maturity may occur in different periods, and it will set an analytical and interpretive trend of the results [46, 47].

Another aspect that may interfere in seroprevalence results of a given sample refers to the chromogen used in immunoenzymatic assays. In addition, when comparing OPD chromogen sensitivity with ABTS, it is possible to find that OPD chromogen is more sensitive and detects antibodies expressing higher optical densities as previously reported [48]. Thus, we used a higher concentration of antigen to sensitize the plate in ELISA with ABTS due to lower detection sensitivity of this chromogen.

In this study, ELISA test with IgG antibodies, ABTS or OPD chromogen, demonstrated the same sensitivity and specificity for toxoplasmosis reagent sera (Table 2). However, it is important to emphasize that values could be the result of the amount of antigen employed in the sensitization of plates, since 10 μg was used in the ELISA test with ABTS chromogen and, in the ELISA test with OPD, only 1 μg was used. This difference in antigen amount may have favored the convergence of results of toxoplasmosis seroprevalence in immunoenzymatic assays.

A careful evaluation of serological tests may result in useful data for the prevention and institution of protocols for the detection of groups at risk, including the IgG avidity test. Transversal studies, as simple as this one, may help in a correct analysis of the results and, accordingly, collaborate for proposals and development of strategies to facilitate the interpretation of diagnosis, and this may result in more reasonable prophylactic measures for controlling this infection.

Soil and consumption of food contaminated with oocysts are considered, in some regions, the major sources of infection by *T. gondii* [49]. However, in the sample of the present study, the relation between these variables and the frequency of seropositive results was not statistically significant. On the other hand, the variable contact with cats outside the university possibly had a more important role, based on a higher frequency of seropositive results among those who had contact with those animals (76.0%) and those who were used to consume raw or undercooked meat (92.0%). Such differences are considered statistically significant, in agreement with previous studies, in which frequent ingestion of raw or undercooked meat and contact with oocysts of infected felines are considered the main risk factors for *T. gondii* infection [49–52].

Concerning gender, no significant differences were noted, which is in agreement with Souza [53], Daguer *et al*. [54] and Sharif *et al*. [55].

## Conclusions

In the present study, the use of 2,2′-azinobis(3-ethylbenzothiazoline-6-sulfonate) (ABTS) and O-phenylenediamine dihydrochloride (OPD) chromogens in immunoenzymatic ELISA assay with adequate quantities of *T. gondii* antigen for each substrate showed the same sensitivity for detection of toxoplasmosis reagent sera (Table 2).

According to the current findings, the cat population within the university is not a risk factor for *T. gondii* infection. Therefore, one may conclude that the most relevant risk factors for toxoplasmosis were contact with cats outside UNESP and eating raw or uncooked meat (Table 4).

The extrapolation of these results to the general population must be carefully considered, since the investigation was conducted on a reduced sample. However, it allows stating the importance of careful and well prepared studies, not only to identify risk factors for toxoplasmosis, but also to adopt preventive measures and offer guidance to at-risk populations. Thus, the implementation of simple cross-sectional studies may provide important epidemiological contribution and establish consistent causal associations, avoiding thereby the development of hypotheses that do not support rigorous questioning.
